# Highly Responsive Mid-Infrared Metamaterial Enhanced Heterostructure Photodetector Formed out of Sintered PbSe/PbS Colloidal Quantum Dots

**DOI:** 10.1021/acsami.2c23050

**Published:** 2023-02-16

**Authors:** Raphael Schwanninger, Stefan M. Koepfli, Olesya Yarema, Alexander Dorodnyy, Maksym Yarema, Annina Moser, Shadi Nashashibi, Yuriy Fedoryshyn, Vanessa Wood, Juerg Leuthold

**Affiliations:** † Institute of Electromagnetic Fields, ETH Zurich, 8092 Zurich, Switzerland; ‡ Institute for Electronics, ETH Zurich, 8092 Zurich, Switzerland

**Keywords:** photodetectors, metamaterial, mid-infrared, quantum dots, heterostructure, PbSe, PbS

## Abstract

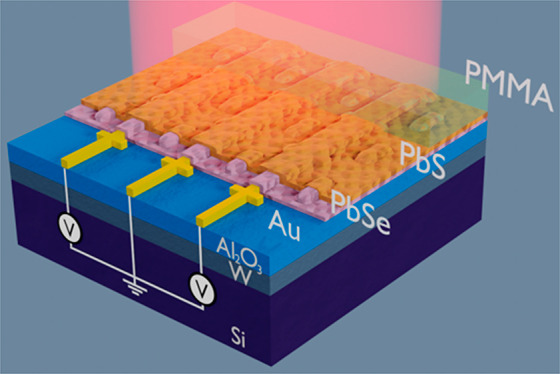

Efficient and simple-to-fabricate light detectors in the mid infrared (MIR) spectral range are of great importance for various applications in existing and emerging technologies. Here, we demonstrate compact and efficient photodetectors operating at room temperature in a wavelength range of 2710–4250 nm with responsivities as high as 375 and 4 A/W. Key to the high performance is the combination of a sintered colloidal quantum dot (CQD) lead selenide (PbSe) and lead sulfide (PbS) heterojunction photoconductor with a metallic metasurface perfect absorber. The combination of this photoconductor stack with the metallic metasurface perfect absorber provides an overall ∼20-fold increase of the responsivity compared against reference sintered PbSe photoconductors. More precisely, the introduction of a PbSe/PbS heterojunction increases the responsivity by a factor of ∼2 and the metallic metasurface enhances the responsivity by an order of magnitude. The metasurface not only enhances the light–matter interaction but also acts as an electrode to the detector. Furthermore, fabrication of our devices relies on simple and inexpensive methods. This is in contrast to most of the currently available (state-of-the-art) MIR photodetectors that rely on rather expensive as well as nontrivial fabrication technologies that often require cooling for efficient operation.

## Introduction

The mid infrared (MIR) electromagnetic spectrum is a wavelength range that is of great importance for various applications and technologies. These applications include gas sensing,^1,2^ thermal imaging,^3^ biosensing,^4^ medical imaging,^5^ spectroscopy, and environmental monitoring.^6^ A key component to make such technologies practical are photodetectors. Thus, the demand for cost-efficient highly responsive detectors operating at room temperature increases steadily. Most of the currently available detectors are based on quantum well structures, III–V compound semiconductors, or mercury–cadmium-telluride alloys such as InSb, In_1–*x*_Ga_*x*_As, and Hg_1–*x*_Cd_*x*_Te.^7,8^ However, detectors fabricated from these materials are quite expensive, require complex fabrication processes, and often need cooling, thus limiting their use for various applications.

Alternatively, lead chalcogenides have been used since the 1950s for MIR photodetection and have recently re-emerged due to their high MIR sensitivity and low cost.^8,9^ PbSe is particularly promising and is one of the most used materials for photodetection in the MIR spectrum due to its low cost and high detectivity at room temperature.^9^ Although PbSe has been used for photodetection for several decades, a full understanding of the photoconduction mechanism is still lacking. Pristine PbSe shows no significant photoresponse in the MIR. However, with a thermal treatment in an oxygen and iodine atmosphere—referred to as sensitization—MIR detection becomes possible.^10−12^ Furthermore, the performance of PbSe detectors depends on which deposition method is used, with the primarily used methods being chemical bath deposition (CBD) and chemical vapor deposition (CVD), which have both their benefits and drawbacks.^9^ CBD is a cheap process where PbSe is grown on a substrate in solution. This deposition technique has the disadvantage that it is challenging to reproduce, it is difficult to achieve thin uniform layers over a large area, and it is limited in that it is substrate dependent.^9^ Contrary to CBD, CVD enables the deposition over large areas, but the material typically exhibits worse photoconduction than CBD-grown PbSe.^9^ An attractive alternative to polycrystalline PbSe are colloidal quantum dots (CQDs). They are substrate independent, absorption tunable, and can be deposited from solution, which makes them a promising candidate for cost-efficient photodetectors;^13−16^ however, so far, they display low quantum efficiency in the MIR.

In recent years, considerable effort has been put into researching strategies to enhance the photoresponse of detectors based on bulk, two-dimensional (2D), and CQD materials. One way to enhance the absorption and therefore the photoresponse are metamaterials. Such artificial materials consist of periodically-arranged, subwavelength structures, which can resonantly couple to incoming electromagnetic (EM) fields. These resonating elements can scatter the EM field into photoactive materials and enhance the photoresponse.^17,18^ Metamaterials can be designed with numerous different material stacks and geometries depending on the desired characteristics such as a narrow or broad band absorption and on the wavelength regime for which they are intended.^17^ And indeed, metamaterials have been repeatedly used to enhance the absorption of photodetectors, but most designs do not take the charge extraction into account. Photogenerated carriers often must travel large distances to the electrodes and charges recombine before they can be collected, which reduces the photoresponse.

An additional way of increasing the photoresponse is by combining different materials in a heterojunction configuration. Such stacks have been realized by combining 2D, bulk, or CQD materials in a planar or vertical fashion.^19,20^ In planar heterojunction configurations, the layers can be arranged to provide photogain. In heterojunctions, electrons and holes are spatially separated at the interface of different materials due to a built-in electric field and one type of charge carrier is transported to electrodes whereas the other is trapped. The trapping of one type of carrier and the transport of the other to the electrodes can result in gain if the trapping lifetime is longer than the transient time.^19^ Such schemes have also been used in photodetectors based on organic materials where the gain resulting from charge trapping is often referred to as photomultiplication.^21,22^ Further, the spatial separation of carriers can result in reduced charge recombination.^19^ Although heterojunctions can enhance the detector performance, they also have drawbacks. Most heterojunction photodetectors are implemented in a vertical configuration, thus transparent electrodes are required, which can be challenging and restricting due to the small number of suited materials. Additionally bulk heterojunctions are often deposited via molecular beam epitaxy, which makes them expensive. Whereas heterojunctions employing 2D materials seem promising, the large area deposition of these materials is still difficult.^23^

In this work, we overcame low photoresponse, expensive and complicated absorber material deposition, and the necessity for low-temperature operation by combining a metamaterial perfect absorber, a heterojunction, and a sintered CQD absorbing layer into one device to demonstrate a highly responsive, simple to fabricate, and compact MIR photoconductor operating at room temperature. We found responsivities as high as 375 A/W at 2712 nm and 4.8 A/W at 4250 nm. The absorber material was fabricated out of NIR absorbing PbSe CQDs, which were surface modified and thermally treated to form a MIR absorbing polycrystalline layer, without the need of sensitization. This deposition method has the possibility of being cost efficient and allows^13−16^ for large area deposition of thin uniform layers with good thickness controllability due to solution processability. In addition, the developed recipe allows for stacking of different materials. This way we can deposit and crystallize a thin PbS layer on top of the crystallized PbSe layer, forming a beneficial heterojunction. The PbSe/PbS layers were finally combined with a metasurface to selectively enhance the absorption in a wavelength range from 2.7 to 4.2 μm. This metasurface was designed in such a way that it allows both absorption enhancement and charge extraction.

## Device Structure and Fabrication

**Figure 1:**
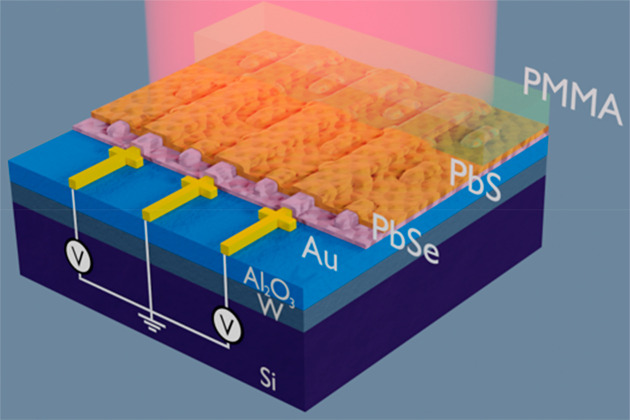
Schematic illustration of metamaterial enhanced heterojunction photodetector with top illumination. The 100 nm tungsten (W), 100 nm alumina (Al_2_O_3_), and 70 nm thick gold (Au) layer form the metasurface, which is used to enhance the absorption and contact the photoconducting heterojunction layer (30 nm PbSe/25 nm PbS). The width of the contact lines and the dipole resonators was 80 nm. The 120 nm PMMA layer is used for passivation.

We developed efficient MIR photoconductors by combining a metasurface with a heterostructure PbSe/PbS absorber stack (Figure 1). The metasurface consists of a metal–insulator–metal stack designed to integrate dipole resonators with an interdigitated electrode structure, which contacts the photoconducting lead chalcogenide layers. Consequently, the generated in-plane dipole field can be efficiently absorbed by the PbSe and only a very thin absorber layer is needed to fabricate highly responsive photodetectors.

To fabricate the envisioned heterojunction metamaterial enhanced photodetectors, we first optimized the sintered colloidal CQDs layers. We then fabricated MIR photodetectors, built from these optimized sintered CQDs layers, and demonstrate their excellent characteristics stemming from the planar PbS/PbSe heterojunction; finally, this layer stack was combined with a metamaterials stack and characterized.

### Optimizing the Deposition and Crystallization of CQD Lead Chalcogenide Absorber Layers

The developed deposition recipe offers various advantages compared to conventional lead chalcogenide deposition methods. It is simple, can be applied to various substrates, it is CMOS, low cost, and results in uniform layers with a surface roughness as low as 3–4 nm. The deposition method is CMOS compatible in the sense that the deposition technique of the CQDs, the used chemicals, and applied annealing temperatures are compatible with standard materials and fabrication processes used for CMOS technology.^24,27^ Furthermore, the low costs arise from the fact that there is no need for expensive and sophisticated deposition infrastructure compared to CVD, making this deposition method attractive for low-cost fabrication and high throughput.^13−16^

PbSe and PbS CQDs were selected as absorber materials. The synthesis of these colloids is well-studied and yields high-quality monodisperse CQDs. Lead chalcogenide CQDs have been successfully employed in the visible and NIR spectral ranges, but devices operating in the MIR have been scarcely reported and often perform worse than their bulk counterparts.^28−33^ In order to combine the deposition advantages of CQDs and the performance of bulk materials, we have developed a liquid-phase spin-coating fabrication recipe, followed by a solid-state surface modification of CQDs (i.e., ligand exchange) and thin-film annealing step. The latter triggers microscale sintering of CQDs layers, enabling bulk-like transport and extended absorption to the MIR.

**Figure 2:**
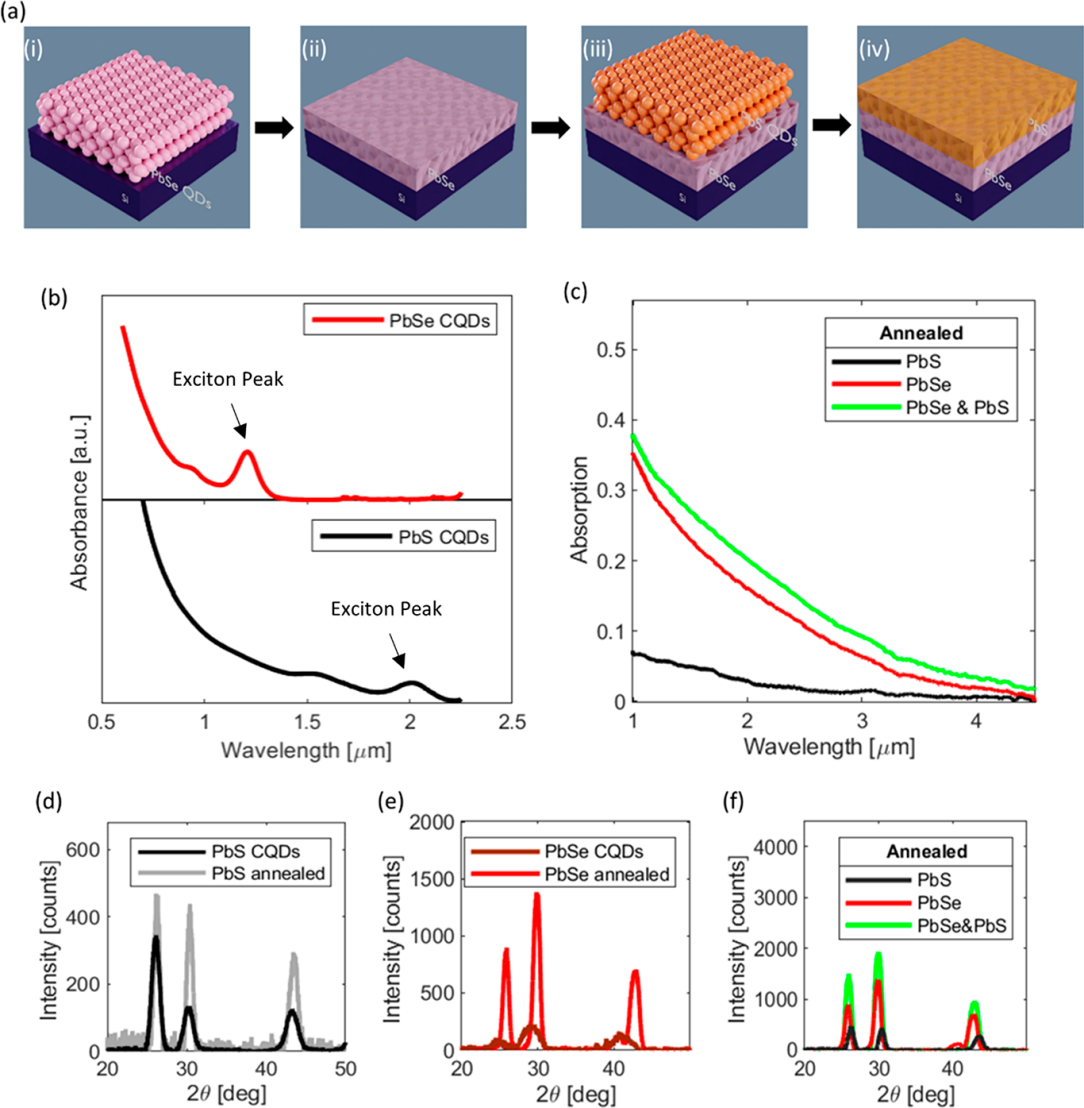
(a) Illustration of the fabrication of the annealed PbSe/PbS bilayer stack layers as used for the GIXRD (grazing incidence X-ray diffraction) characterization. (i) Solution deposited PbSe CQDs on Si substrate after annealing, at 130 °C for 1 min. (ii) Sintered PbSe layer after elevated temperature annealing at 310 °C for 1 min. (iii) Solution deposited PbS CQDs on the sintered PbSe CQD layer. (iv) Sintered PbSe/PbS bilayer stack after second elevated temperature annealing, 310 °C for 1 min. (b) UV–vis absorbance spectra, in arbitrary units, of CQDs in solution, showing absorbance peaks resulting from the quantum confinement and no absorbance for wavelengths larger than 2 μm. This plot shows the general shape of the absorption curve of the CQDS in solution and is not an absolute measure. (c) FTIR absorption spectra of the annealed PbSe and PbS CQD and of the stacked and annealed PbSe/PbS layers on sapphire substrates. The annealing results in the sintering of the individual CQDs into larger crystal domains, which results in the loss of excitonic peaks. The spectrum now approaches a bulk spectrum with absorption up to the MIR. (d and e) XRD patterns of CQDs and annealed layers. (f) XRD pattern of annealed PbSe, PbS, and stacked PbSe/PbS layer.

More precisely, the PbSe CQDs in octane were spin-coated on Si substrate with a native oxide on top. After the deposition, the nonvolatile oleic acid ligands were exchanged for short and more volatile ethanedithiol (EDT) molecules. Afterward, the substrate with the CQD thin film was placed on a hot plate at 130 °C for 1 min to evaporate the excess solvents and EDT ligands. This deposition cycle was repeated until the desired thickness of the CQD layer was reached. Finally, the whole stack was annealed at 310 °C to sinter the CQDs and form a bulk-like polycrystalline layer. Then, PbS CQDs were either deposited onto Si substrates or on a previously annealed PbSe CQD layer, followed by the same spin-coating, ligand-exchange, and annealing procedure as for the PbSe CQDs. Figure 2a provides a schematic of the deposition and annealing process. A detailed description of the synthesis of the used PbSe and PbS CQDs with diameters of 4 and 8 nm is given in the Supporting Information Section 1.

The absorption spectra of the pristine CQDs in solution have a distinct exciton peak at a wavelength of 1200 nm for PbSe and 2010 nm for PbS and no absorption beyond these wavelengths (Figure 2b). The local exciton absorption peak and the lack of an absorption beyond 1200 and 2010 nm, compared to the bulk materials, which absorb further in the MIR, are clear indicators that electron and hole confinement is present before the annealing. The excitonic peaks and the blue-shifted absorption spectra occur when bound electron–hole pairs (exciton) are confined to a space smaller than their exciton radius. The confinement leads to discretization of the quasi-continuous valence and conduction bands. Thus, the absorption spectra can be used to verify if the colloidal quantum dots sinter into larger crystal domains and lose their confinement.^34−36^

When the CQD layers are annealed, they sinter into larger crystal domains and the absorption becomes more bulk like. The electron and hole pairs are now located in a space larger than their exciton radius; as a result, no discrete energy states larger than the bulk band gap of PbS and PbSe are present. As a consequence, the absorption extends after the annealing and sintering to the MIR. Figure 2c shows the absorption spectra of the annealed thin films, it can be seen that the exciton peaks due to the quantum confined have disappeared and the sintered layers absorb in the MIR. Furthermore, the measurements show the PbSe layers to be stronger absorbing than the PbS layers. This is similar to what can also be seen from bulk PbSe and PbS.^37^ The deposited PbSe layer was ∼30 nm, and the PbS layer was ∼25 nm thick. The thickness of these layer was acquired with atomic force microscopy (AFM) measurements; details can be found in the Experimental Methods section.

We additionally did grazing incidence X-ray diffraction (GIXRD) characterizations to observe the changes of the crystallinity upon annealing. Parts d and e of Figure 2 show the GIXRD plots of the PbS and PbSe CQDs before and after the annealing step. It is clearly visible that the peaks become narrower and more pronounced after annealing at 310 °C, which indicates the sintering of CQDs into larger crystal domains. GIXRD characterizations at various annealing times revealed interesting trends: While the crystallinity of PbSe CQD thin film decreases with increasing annealing time, the PbS layer shows the opposite trend (Figure S2a,b. These trends were determined since the intensity of the characteristic peaks of PbS in the XRD pattern increased with the annealing time and the intensity of the PbSe peak decreased. In addition to GIXRD, we characterized the CQD thin films using AFM, which shows that the CQD layers crystallize upon annealing into grains of 30–50 nm (Figure S3).

We performed additional GIXRD measurements with single layer PbS and PbSe and with the annealed PbSe/PbS stack to show that the annealed PbSe/PbS bilayer remains a stack of two different materials and does not form a homogeneous ternary PbS_*x*_Se_1–*x*_ compound (see Figure 2f). The shape of the two single materials PbS (black) and PbSe (red) sum up to the pattern of the stacked PbSe and PbS material (green), indicating that the materials do not form a single compound. A more detailed plot showing this can be found in Figure S2c. In Figure S2c in the Supporting Information, it can be seen that the slight offset between the PbS and PbSe peak position translates to a minor asymmetry of the intensity peaks of the stacked PbSe/PbS XRD pattern.

### PbSe/PbS Heterojunction Photoconductors

After the development of the deposition and annealing recipes, we carried out an electro-optical characterization of the sintered CQDs to investigate their MIR photoresponse and show the advantages of the heterojunction, which resulted in ∼2-fold increase of the photoresponse compared to pristine PbSe at a wavelength of 2710 nm. The increased photoresponse of the bilayer devices is a result of trap assisted gain, which is discussed in more detail below.

**Figure 3:**
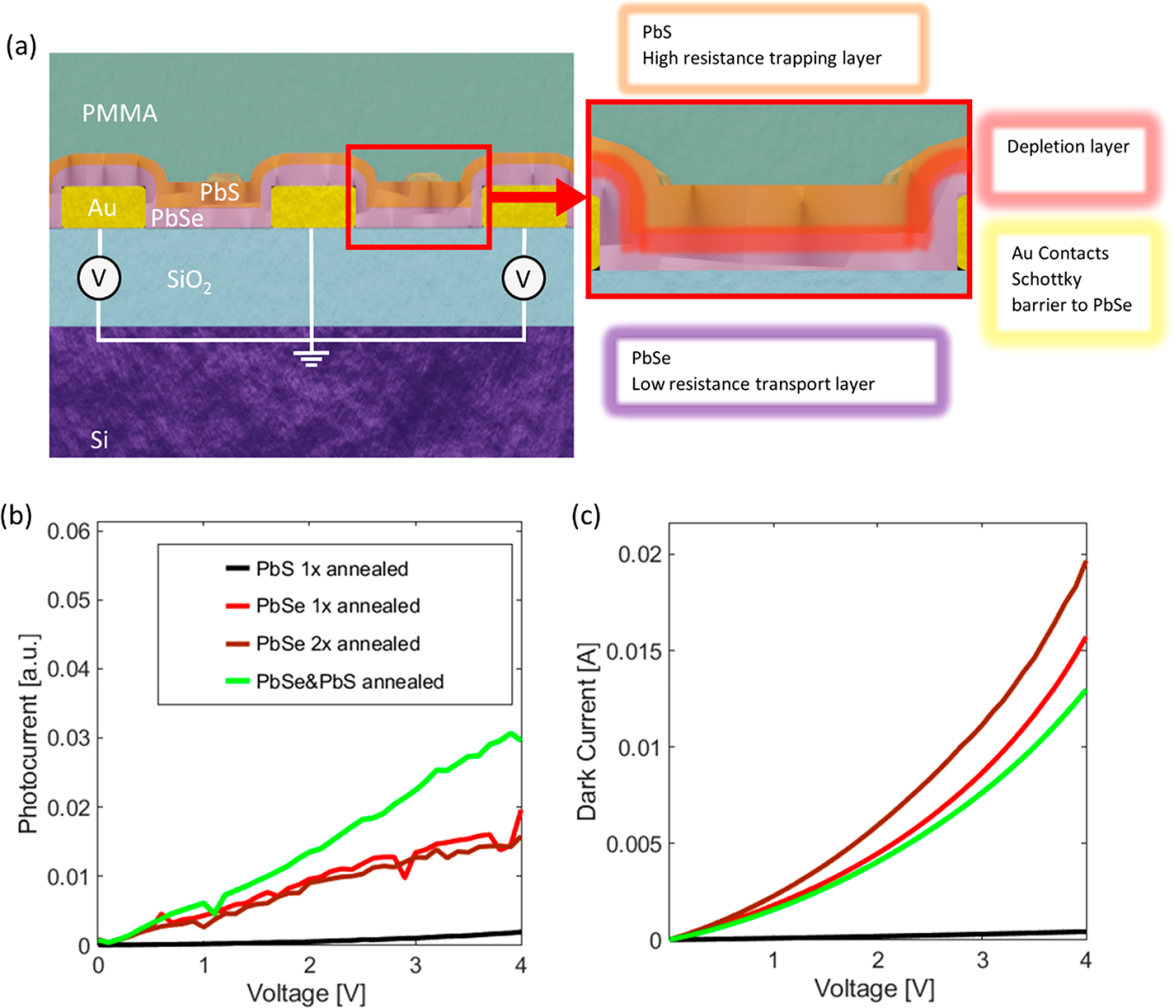
(a) Schematic cross-section of bilayer photodetector with zoom-in and function description. The photodetector consists of gold (Au) interdigitated electrodes on top of a silicon (Si) substrate with a thermally grown silicon oxide (Si_2_O_3_) and a PbSe/PbS heterojunction photoconducting layer, which is passivated by a PMMA layer. The zoom-in shows the PbSe layer, which forms a Schottky type contact to the Au electrodes and is responsible for the main absorption and current transport. The high-resistance PbS layer forms a heterojunction with the crystal grains of the PbSe layer. A depletion layer is formed in-between (red layer). (b) Voltage dependent photocurrent of the photodetectors with annealed photoconducting layers under identical illumination conditions at a wavelength of 2710 nm. The PbSe layers (red, brown) show a ∼10 times greater response than PbS (black). The implementation of the heterojunction leads to a further ∼2-fold increase of the response (green). (c) Dark current of the photodetectors with annealed photoconducting layers. Illustrating the larger conductivity of the PbSe layers (red, brown, green) compared to that of the PbS layer (black).

To characterize the sintered CQDs, we fabricated simple photoconductor structures consisting of an Au interdigitated finger structure on top of a Si substrate with a thermally grown SiO_2_ layer. The characterized photoconductor materials are the annealed PbSe/PbS stack and for referencing the pristine PbSe and PbS. The PbSe photoconductor was furthermore annealed twice in order to guarantee that it undergoes the same number of annealing steps as the PbSe/PbS stack. After the deposition of the photoactive materials, a PMMA layer was deposited on top to prevent oxidation. Each of the deposited layers was 25–30 nm thick; consequently, the PbSe/PbS stack was 50–60 nm thick. A schematic cross section of such a device with a PbSe and PbS layer can be seen in Figure 3a, and detailed description of the device geometry can be found in the Experimental Methods section. The photoconductors were characterized at a wavelength of 2710 nm in a custom-built setup (Figure S4).

Figure 3b shows the resulting photocurrent (under identical illumination conditions) of the different photoconductor materials and their combination. PbS exhibits the lowest photoresponse, whereas the once and twice annealed PbSe layers display a ∼10-fold increase in photocurrent. The difference of the photoresponse can be attributed to the properties of the materials, because PbS is absorbing less light than PbSe, as shown in Figure 2c. Furthermore, the conductivity of the PbS layer is lower than for the PbSe, which results in lower currents, as shown in Figure 3c. The lower conductivity of PbS may be partially the result of a lower electron and hole mobility μ_e_ and μ_h_. A lower electron and hole mobility of PbS have been reported in the literature (μ_e_ ∼ 600 cm^2^ V^–1^ s^–1^ and μ_h_ ∼ 700 cm ^2^ V^–1^ s^–1^) when compared to those of PbSe (μ_e_ ∼ 1200 cm^2^ V^–1^ s^–1^ and μ_h_ ∼ 1000 cm^2^ V^–1^ s^–1^).^38,39^ Also, due to the lower mobility, it is expected that less photogenerated carriers can be extracted before they recombine resulting in a lower photoresponse.

We want to point out that the deposited PbSe was not sensitized and is still photoconducting in the MIR wavelength regime. A discussion on the photoresponse of the annealed PbSe CQDs can be found in the Supporting Information Section 5.

The benefit of implementing a bilayer stack can be seen by the ∼2-fold increase of the photocurrent compared to single layer PbSe devices (see Figure 3b). Notably, the photocurrent is larger than the sum of contributions of the individual materials on their own. This increase of the photocurrent is attributed to photogain obtained by the PbSe/PbS heterojunction.

The origin of the observed photogain can be understood as follows: Photons are primarily absorbed in PbSe, creating free electrons and holes. These carriers are then separated by the built-in field of the PbSe/PbS heterostructure interface. We assume that photogenerated electrons in the PbSe layer are transported to the PbSe/PbS interface where they are trapped, whereas holes are transported to the Au electrodes as a result of the externally applied electric field between the gold electrodes. The assumption of the separation of the photogenerated carriers is based on the bulk conduction and valence band structure resulting from the electron affinities χ and band gaps E_g_ of PbSe and PbS (χ_PbSe, bulk_ ≈ 4.7 eV, E_g, PbSe_ ≈ 0.27 eV χ_PbS, bulk_ ≈ 4.55 eV, E_g,PbS_ ≈ 0.4 eV).^39−42^ Further, EDT treatment of PbSe and PbS is known to result in p-type CQD layers.^43,44^ It is therefore assumed that the annealed colloidal QDs still exhibit p-type behavior after the annealing.

Similar trapping schemes have been applied with various different photoactive materials and device structures.^43,45−48^ If the trapping lifetime *t*_lifetime_ of the photogenerated electrons is larger than the transient time *t*_transit_ of the holes to the electrode, gain occurs since the charge neutrality condition must apply. The theoretical gain *G*_th_ of a photoconductor can thus be defined as *G*_th_ = *t*_lifetime_/*t*_transit_. It is desirable that the charge transport layer has a high mobility since the transit time can be expressed as *t*_transit_ = *L*^2^/*μV* Therefore, the mobility can directly influence the gain and with it the photoresponse.^19,49−51^ The absorber stack and the properties of each material can be seen in the zoom-in of Figure 3a.

The formation of a heterostructure is thus important for obtaining photogain. Various accounts on the increase of responsivities due to the presence of a heterojunction have already been given for such planar configurations.^43,52−55^ Indication for the presence of a heterojunction between the PbSe and PbS can be derived from Figure 3c. The figure shows the dark current between the Au electrodes for a PbSe layer stack (brown and red). When the dark current is measured for the combined layer stack of PbSe/PbS, one finds a lower dark current. This can be explained by the built-in field that is formed at the interface due to carrier depletion. This depletion layer reduces the cross-section through which the current is flowing along the lateral direction, therefore increasing the overall resistance, which leads to the lower current. A further discussion on the formation of the heterostructure can be found in the Supporting Information Section 6.

### Passive Characterization of PbSe/PbS Metamaterial Photodetectors

**Figure 4:**
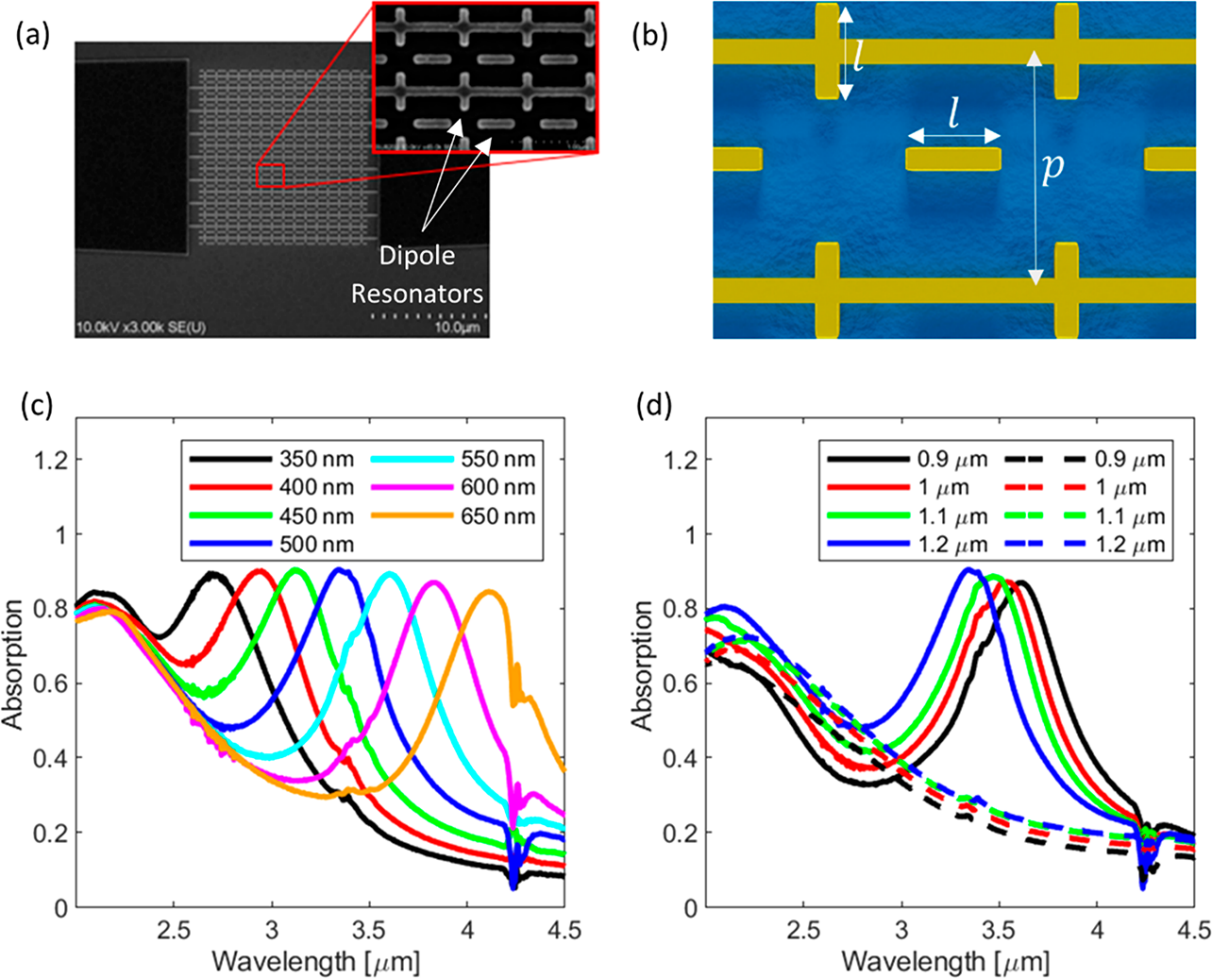
(a) SEM image of the metamaterial design showing the Au top layer with the freestanding and interconnected resonating elements. (b) Top view of the designed metamaterial with the contact lines and dipole resonators including the design parameters. (c) Passive absorption spectra of metamaterial detector. The resonator lengths *l* were varied and the period was kept constant at *p* = 1.2 μm. The absorption peaks shift toward larger wavelengths when increasing the dipole resonator length *l*. (d) Passive absorption spectra of metamaterial detectors. Here, the periods *p* was varied but the resonator length was kept at *l* = 500 nm. A blue shift of the resonance peak with increasing period can be observed. The dashed lines show the absorption spectra of devices without any dipole resonators as a reference.

Lastly, the MIR photosensitive bilayer stack was combined with a metallic metamaterial to selectively increase the absorption. The metamaterial consists out of a metal–insulator–metal stack and does not exhibit any photoresponse without the photoactive PbSe/PbS layer. With the addition of the metamaterial, near unity absorption has been achieved. A scanning electron micrograph and the schematic of the fabricated metamaterial are shown in Figure 4a,b. This structure was covered with the 30 nm PbSe and 25 nm thick PbS bilayer stack. Finally, a 120 nm thick PMMA layer was spin-coated as passivation (see Figure 1).

The metamaterial layer stack consists of a tungsten backplane, an alumina spacer layer, and the gold resonator layer. The resonators are constructed of dipole antennas that are partially interconnected with contact lines. These dipoles act like a deconstructed cross resonator metamaterial perfect absorber and couple to different polarization states of incoming light.^18,25,56,57^ A SEM image with indications of the dipole resonators as well as a partial schematic of the metamaterial can be found in Figure 4a,b. The resonance is hardly perturbed by the connection line and the metamaterial generates an in-plane dipole field that can be efficiently absorbed by an absorbing layer placed on top. A detailed discussion on the shown metamaterial can be found in a previously published work.^57^

The narrow absorption peaks resulting from the metamaterial can be shifted across a wide wavelength range by changing the dimensions of the metasurface. The parameters used to tune the absorption peak position are the length *l* of the resonators and the period *p* of the square unit cell shown in Figure 4b. When the resonator length is increased the absorption peak is shifted to longer wavelengths, whereas the peaks are shifted to shorter wavelength by increasing the period between the contact lines, which is shown by the measured plots in Figure 4c,d. With the selected design parameters, an absorption for unpolarized light of approximately 90% could be reached. Further, the absorption is nearly polarization independent due to different orientation of the dipole antennas and does not drop below 75% while reaching 98% for an ideal polarization state (see Figure S7). This indicates that nearly perfect absorption could be achieved by slightly correcting the length of the dipoles.

This metamaterial design offers an additional feature since two parameters can be used to tune the absorption peak position. It is possible to tune the photocarrier extraction efficiency without changing the absorption spectra by reducing the period between the contacts and at the same time reducing the resonator length.

## Results

Followed by the passive absorption characterization, metamaterial devices were selected with absorption peaks at wavelengths of 2730, 3250, 4000, and 4250 nm and electro-optically characterized at these wavelengths. The passive absorption spectra of the selected devices are shown in Figure S8. Furthermore, the geometry data of the metamaterial designs can be found in Table S1 of the Supporting Information. Subsequently, the electro-optical characterizations were carried out with either a laser source at 2710 nm or a blackbody light source, which was filtered by bandpass filters. The photoresponse and the noise characterizations were performed with a lock-in-amplifier. All of the results have been obtained for a device with an absorption peak at a wavelength 2730 nm—unless for Figure 6, where device with absorption peaks close to the used characterization wavelengths are compared. Additional measurements and an illustration of the measurement setup can be found in the Supporting Information Sections 4 and 9.

**Figure 5:**
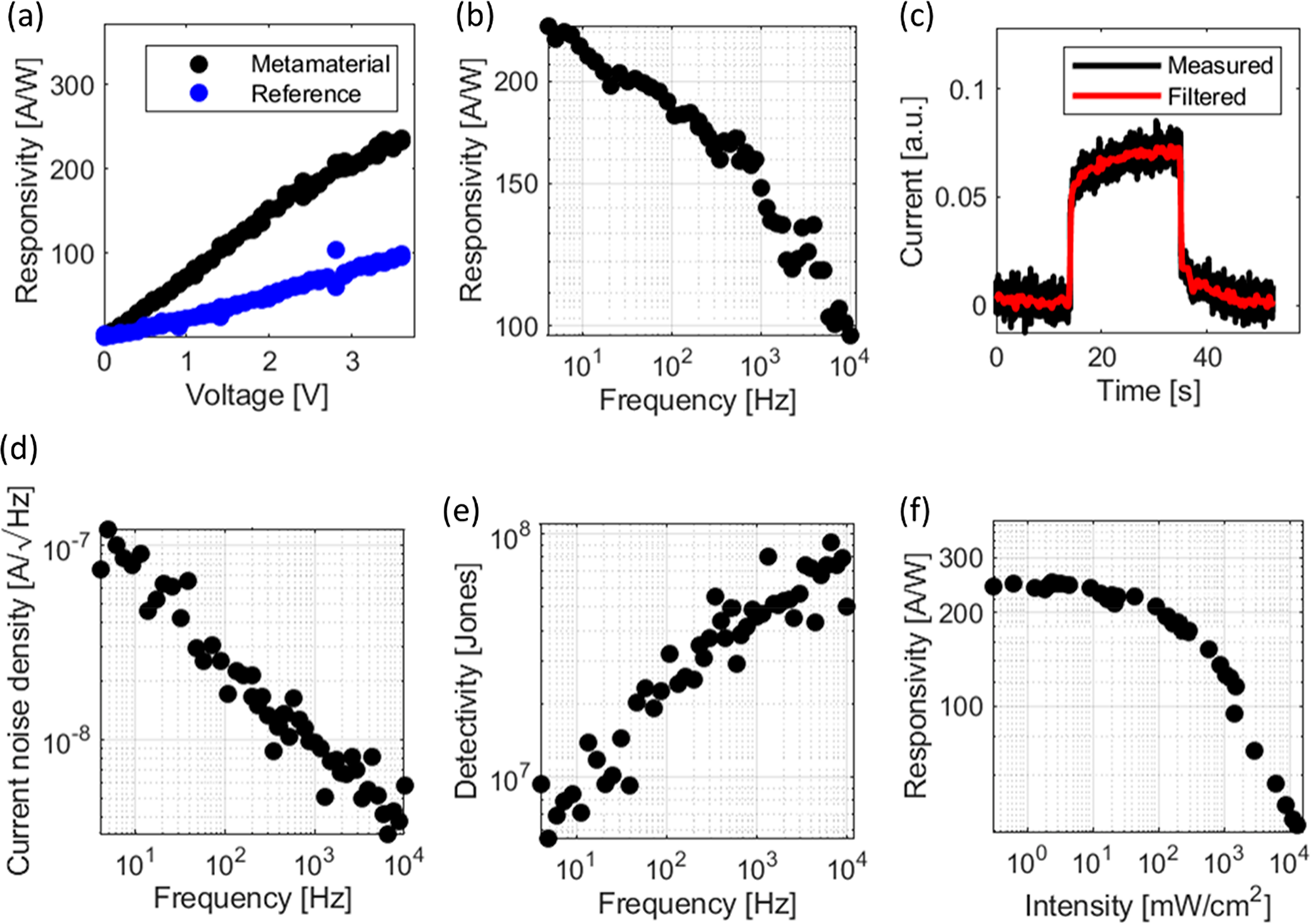
Electro-optical characterization of the detector. (a) Voltage dependent responsivity at 4 Hz modulation frequency of a metamaterial and comparison with an identical reference device without dipole resonators. The metamaterial device clearly outperforms the reference device. (b) Frequency dependent responsivity, 4 Hz–10 kHz. (c) Photoresponse measured over a long period of time. (d) Current noise spectral density. (e) Frequency dependent detectivity. (f) Intensity dependent responsivity (All measurements were performed on a metamaterial enhanced PbSe/PbS heterojunction photodetector with a resonator length of l = 350 nm and a contact spacing of *p* = 1 μm). The reference device had no resonating elements and a contact spacing of *p* = 1 μm. The measurements under illumination were carried out at a wavelength of 2710 nm.

In Figure 5a, the voltage dependent responsivity of a metamaterial photoconductor and a photoconductor without any dipole resonator (see Figure 4a for indication of the dipole resonators), referred to as reference, is shown. Such reference devices were fabricated on the same chip as the metamaterial photoconductors. They consist of 70 nm thick and 80 nm wide Au contact lines spaced with the period *p*, which are covered by the photoactive PbSe/PbS heterojunction and a PMMA layer. The reference devices and the metamaterial devices had the same active area of 30 × 30 μm^2^. The metamaterial detector exhibits a ∼2-fold increase in responsivity at a wavelength of 2710 nm compared to the reference detector. This comparison clearly demonstrates the absorption enhancement due to the metamaterial.

Frequency response measurement in the range from 4 Hz to 10 kHz has been performed (see Figure 5b). The frequency range was limited by the mechanical chopper used to modulate the light source. A maximum response of ∼250 A/W was found at a frequency of 4 Hz. It can be seen from the trend of the responsivity that the responsivity could be higher if measured at frequencies lower than 4 Hz. The maximum responsivity was found by measuring the steady-state photoresponse, (Figure 5c) and scaling it with the measured responsivities from Figure 5b. A maximum responsivity of ∼375A/W was found (details on the measurement are given in the Supporting Information Section 7). Figure 5c was also used to extract the rise and fall times of τ_rise_ = 4.27 s and τ_fall_ = 5.25 s. These long time constants can be often observed in CQD based devices and are attributed to long-lived traps.^30,58,59^ A comparison of recently published works and this work with respect to the responsivity can be found in the Supporting Information Section 10.

While carrier traps can lead to increased responsivity, the trapping and detrapping process also leads to an increase of the current noise.^60−62^ We expect this effect to be the origin of the large noise currents (see Figure 5d). Due to this large noise current, the specific detectivity is limited to ∼1 × 10^8^ Jones, as shown in Figure 5e. The maximum detectivity is found for higher modulation frequencies despite of the responsivity decreasing with frequency. This can be understood by the fact that the noise current decreases with frequency at a larger scale, a finding often observed for PbSe based detectors.^31^

The metamaterial detector was finally characterized with respect to the incoming light intensity. It can be seen in Figure 5f that the detector operates over a large intensity regime.

**Figure 6:**
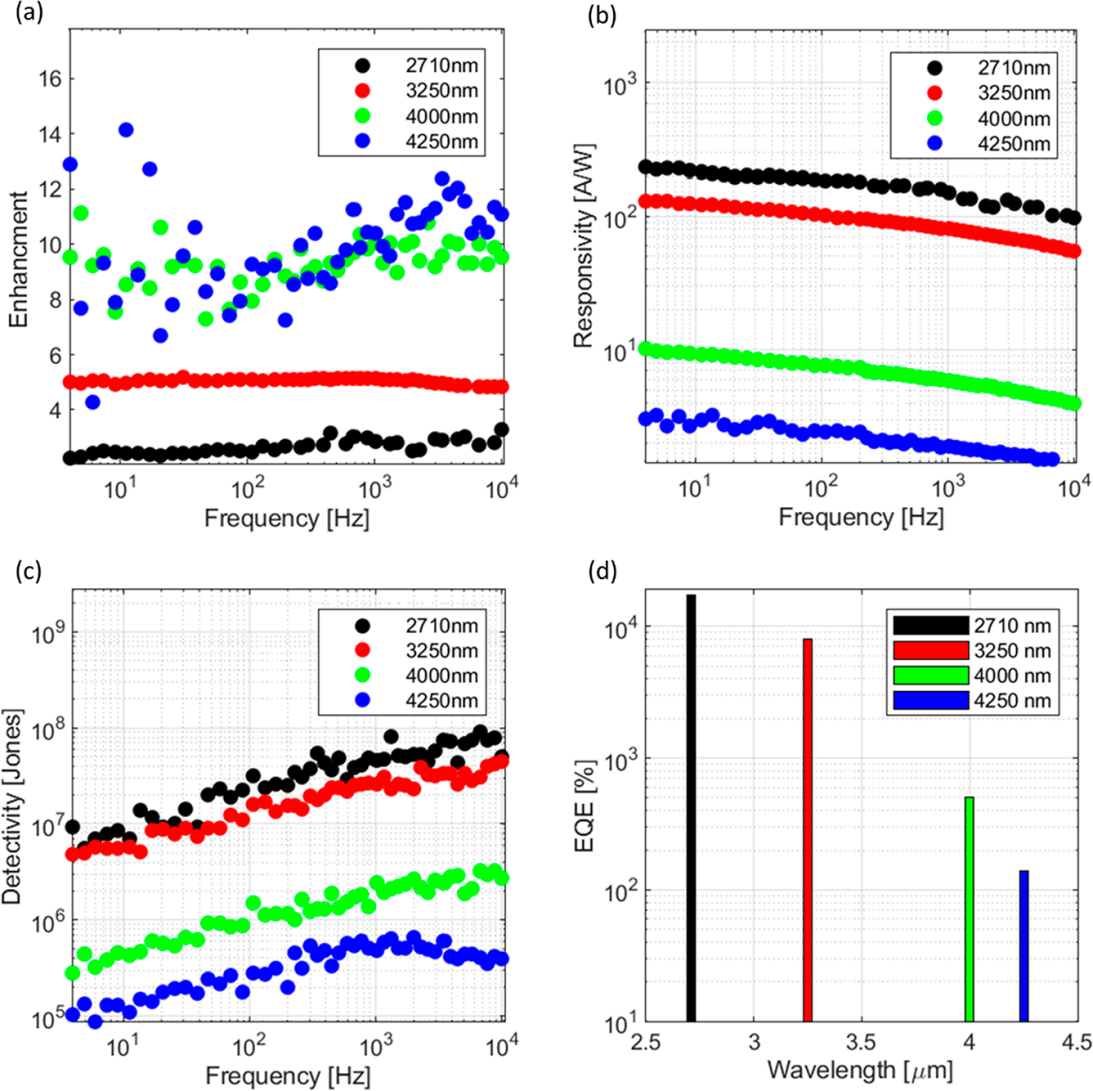
(a) Enhancement (ratio of responsivity of a metamaterial enhanced photodetector against a reference photodetector without resonating elements) vs frequency for different metamaterial photodetectors characterized at different wavelengths. An increase of the enhancement with the wavelength can be observed. (b) Responsivity vs frequency for different metamaterial detectors characterized at different wavelengths. (c) Detectivity of different metamaterial detectors measured characterized at different wavelengths. (d) EQE of four different metamaterial detectors characterized at four different wavelengths. The maximum responsivity was assumed.

To show the benefits of the metamaterial, the fabricated detectors were compared to reference devices. The reference devices consist of an interdigitated finger structure spaced with the same pitch *p* as the metasurface but without any dipole resonators. The ratio between the responsivities of the metamaterial and the reference detectors was used as a figure of merit and is referred to as enhancement. The enhancement for different detectors at different wavelengths is shown in Figure 6a. From this plot, it can be seen that the enhancement increases with wavelength. This can be understood as follows. The absorption is already quite good for shorter wavelengths (see Figure 2c). And indeed, the responsivity is quite large for shorter wavelengths as can also be seen from Figure 6b. Therefore, there is little to be won from the metamaterial enhancement. Yet, the enhancement is more efficient at longer wavelength, where the absorption is smaller, and a resonant enhancement effectively provides a longer interaction with the absorber and therefore an enhancement for the overall absorption.

The enhancement increase for larger wavelengths can also be seen when comparing the absorption spectra of the metamaterials with the corresponding reference device absorption (Figure 4c,d).

In Figure 6b, the frequency dependent responsivity is shown for the different metamaterial devices and wavelengths. The overall decrease of the responsivity with increasing frequency is present for all devices and independent of the metamaterial geometry and can therefore be attributed to the material properties of the photoconductors. Furthermore, in Figure 6c, the detectivity is shown. As expected, it scales in the same manner as the responsivity. The responsivity could be additionally used to estimate the external quantum efficiency (EQE) according to the relation .^26,49^

## Conclusion

In conclusion, we presented a metamaterial enhanced MIR photoconductor operating at room temperature with responsivities as high as 375 A/W at a wavelength of 2710 nm and 4.8 A/W at 4250 nm. These values have been reached by systematically enhancing the device performance by implementing a PbSe/PbS heterojunction absorber stack with a metasurface absorption enhancement scheme. The absorber stack has been fabricated out of NIR absorbing CQDs, which were deposited from solution and then annealed into a polycrystalline like layers. With this method, it is possible to combine the simple and cost-efficient deposition of CQDs, such as spin-coating, with superior bulk MIR absorption properties of lead chalcogenides.

We have shown that the developed deposition method can be used to fabricate MIR photoconducting PbSe layers without the need of sensitization, which is a necessary step for CBD or CVD PbSe to show a relevant MIR photoresponse. Furthermore, the dark current could be decreased and the photoresponse increased ∼2-fold by realizing a PbSe/PbS bilayer stack, which forms a heterojunction on a crystal grain basis, compared to a single layer PbSe device. This bilayer stack was last combined with a metallic metasurface perfect absorber. The chosen metamaterial design allowed to selectively enhance the spectral absorption of the detectors ranging from 2.7 to 4.2 μm. An overall responsivity increases by up to a factor ∼20 has been achieved, demonstrating the benefit of the enhancement schemes. The combination of the developed deposition recipe and the metasurface has the potential to satisfy the growing demand for highly responsive MIR photodetectors fabricated by cheap and simple means.

## Experimental Methods

### CQD Synthesis

Information on the CQD synthesis and transmission electron microscopy can be found in the Supporting Information Section 1.

### CQD Deposition and Annealing

All the following deposition and annealing steps were carried out in an N_2_ atmosphere. The in hexane dispersed CQDs were purified by three cycles of precipitation and redispersion by using centrifugation and anhydrous acetone/hexane as nonsolvent/solvent. After the third purification cycle, the CQDs were dissolved in anhydrous octane, PbSe at a concentration of 12 mg/mL, and PbS at a concentration of 22 mg/mL. The CQDs were spin-coated in a layer-by-layer fashion. CQDs solution was dropped onto the substrate of choice and spun at 2000 rpm for 30 s; then, they were soaked in a 1 vol % EDT acetonitrile solution to achieve a ligand exchange, followed by three rinsing steps. The CQDs were annealed after each spin-coating cycle at 130 °C for 1 min. After the CQD deposition, the CQDs were annealed for 1 min at 310 °C. The bilayers were achieved by depositing the PbS on top of an annealed PbSe followed by the identical annealing procedure as described. This deposition resulted in 25–30 nm thick layers for both PbS and PbSe

### XRD Characterization

The absorber materials were deposited on Si substrates, after which they were placed in a Rigaku SmartLab 9KW XRD-diffractometer for characterization.

### UV–Vis Extinction Measurements

The absorption measurements of the CQDs in solution were carried out with an Agilent Carry 5000. The PbSe and PbS CQDs were dispersed in trichloroethylene for these measurements.

### FTIR Characterization

#### PbSe/PbS Absorption Measurements

The absorber materials were deposited on sapphire windows and characterized with a Bruker vertex 70 FT-IR spectrometer in a transmission measurement configuration.

#### Metamaterial Absorption Measurements

The metamaterial photoconductors were placed in a custom-built setup (Figure S4) and characterized with an Arc Optics FTIR-Rocket spectrometer in a reflection measurement configuration.

### Device Fabrication

#### Interdigitated Finger Structure Photoconductor

The finger structures for the photoconductors were fabricated by a standard photolithography, e-beam evaporation, lift-off process on Si substrates with a thermal grown 200 nm thick SiO_2_ layer. The evaporated Au layer was 70 nm thick and the contacts of the photoconductors were 2 μm wide and 2 μm spaced, resulting in an overall device size of 66 × 20 μm^2^. These structures were covered with the different absorber materials, and finally, a 120 nm thick PMMA was spun on top as an oxidation protection.

#### Metamaterial Device Fabrication

For the metamaterial devices, a 100 nm thick tungsten (W) backplane was sputtered onto Si substrates, which was then covered by 100 nm ALD grown alumina (Al_2_O_3_). The 70 nm thick gold (Au) top resonator structure was fabricated by a standard e-beam lithography, e-beam evaporation, lift-off process. The PbSe layer deposited on to the metasurface was 30 nm, and the PbS layer was 25 nm thick. An oxidation protection of 120 nm of PMMA was spun on the CQDs. The fabricated devices were 30 × 30 μm^2^ in size.

### E/O Characterization

An illustration of the optical setup used can be seen in Figure S4. For the electrical characterization, a Keysight B2902A SMU was used as voltage source and the responsivity and noise measurements were carried out with a lock-in amplifier Zurich Instruments MFLI. A calibrated powermeter, Gentec-EO TH5B-BL-DZ-D0, was used to determine the incoming power on the device.

### Thickness Characterization of Annealed QD Layers

The thickness of the annealed layers was characterized with an AFM. The QDs were deposited onto a Si substrate and partially removed before annealing. After the annealing, the substrate was characterized with an AFM, which allowed us to measure the height difference between the substrate with and without annealed QDs.

## Supporting Information Available

Discussions of CQD synthesis, origin of the photoconductivity in the annealed PbSe CQD layers, presences of a heterojunction, time dependent photoresponse, and comparison of photodetectors, figures of TEM images, XRD spectra, AFM images, optical setup used for the electro-optical characterization, maximum current normalized dark current, time dependent photoresponse, polarization dependent passive absorption, passive absorption spectra, dark *I*–*V* measurements, and responsivity comparison of published works and this work with references, and tables of metamaterial parameters and wavelengths at which they were characterized and comparison of this work and representative recent publications (PDF)
