# Supplementary material for: Highly Responsive Mid-Infrared Metamaterial Enhanced Heterostructure Photodetector Formed out of Sintered PbSe/PbS Colloidal Quantum Dots
Source: ACS Appl Mater Interfaces. 2023 Feb 16;15(8):10847–57. doi: 10.1021/acsami.2c23050 (PMC9982815; doi:10.1021/acsami.2c23050)
Supplement: Supplementary file 1 — am2c23050_si_001.pdf [file am2c23050_si_001.pdf]

Supporting Information

Highly Responsive Mid-Infrared Metamaterial Enhanced Heterostructure Photodetector Formed out of Sintered PbSe/PbS Colloidal Quantum Dots

Raphael Schwanninger [a)], Stefan M. Koepfli [a)], Olesya Yarema [b)], Alexander Dorodnyy [a)], Maksym Yarema [b)], Annina Moser [b)], Shadi Nashashibi [a)], Yuriy Fedoryshyn [a)], Vanessa Wood [b)], Juerg Leuthold* [a)]

a) Institute of Electromagnetic Fields, ETH Zurich, 8092 Zurich, Switzerland
b) Institute for Electronics, ETH Zurich, 8092 Zurich, Switzerland

* Corresponding: Author Juerg Leuthold − *Institute of Electromagnetic Fields*, ETH Zurich, 8092 Zurich, Switzerland; Email: leuthold@ethz.ch

1) CQD Synthesis

Synthesis of PbS Nanocrystals

In a typical synthesis of 8 nm PbS nanocrystals[1], we mix 1.8 g of PbO with 60 ml of oleic acid and 20 ml of 1-octadecene in the three-neck flask. The reactor is then connected to the Schlenk line set-up and heated to 150°C for 1 hour in vacuum. During this time, the mixture is purified from oxygen and water residues, while simultaneously PbO and oleic acid react forming Lead (II) oleate, a precursor of Pb. Afterwards, the reactor is put under a nitrogen stream (1 bar), while the temperature is kept at 150°C. At these conditions, a sulfur precursor mixture, consisting of 0.84 ml of hexamethyldisilthiane and 40 ml of 1-octadecene, is introduced to the reaction via fast injection. The mixture turns brown within a few seconds, indicating a formation of PbS nanocrystals.

To achieve a good size distribution of PbS nanocrystals, the reaction is taken through a optimized temperature profile, namely (i) a natural cooling to 120°C during the first minute of reaction; (ii) slower cooling to 100°C within the next 3 minutes; (iii) annealing at 100°C for 5 minutes; (iv) fast cooling to room temperature to terminate the reaction and mass transfer processes.

As synthesized nanocrystals are then transferred air free to the glovebox and purified by the addition of hexane and ethanol solvents. This step is followed by centrifugation, the overall purification cycle is repeated 3 times.

Synthesis of PbSe Nanocrystals

Synthesis of PbSe nanocrystals is derived from the PbS synthesis above, replacing the sulfur precursor with trioctylphosphine selenide and adding diphenylphosphine as nucleation promoter[2].

In a typical synthesis of 4 nm PbSe nanocrystals, we mix 2.2 g of PbO with 7.86 ml of oleic acid and 34.26 ml of 1-octadecene and heat this mixture at 150°C and vacuum to form Lead (II) oleate. Afterwards, the injection mixture of 30 ml of 1M trioctylphosphine selenide and 0.2 ml of diphenylphosphine is added, triggering the formation of PbSe nanocrystals. The reaction time is shortened to 30 seconds, after what the reaction is terminated by fast cooling to the room temperature. Post-synthetic purification of PbSe nanocrystals is carried out in complete analogy to the PbS synthesis above.

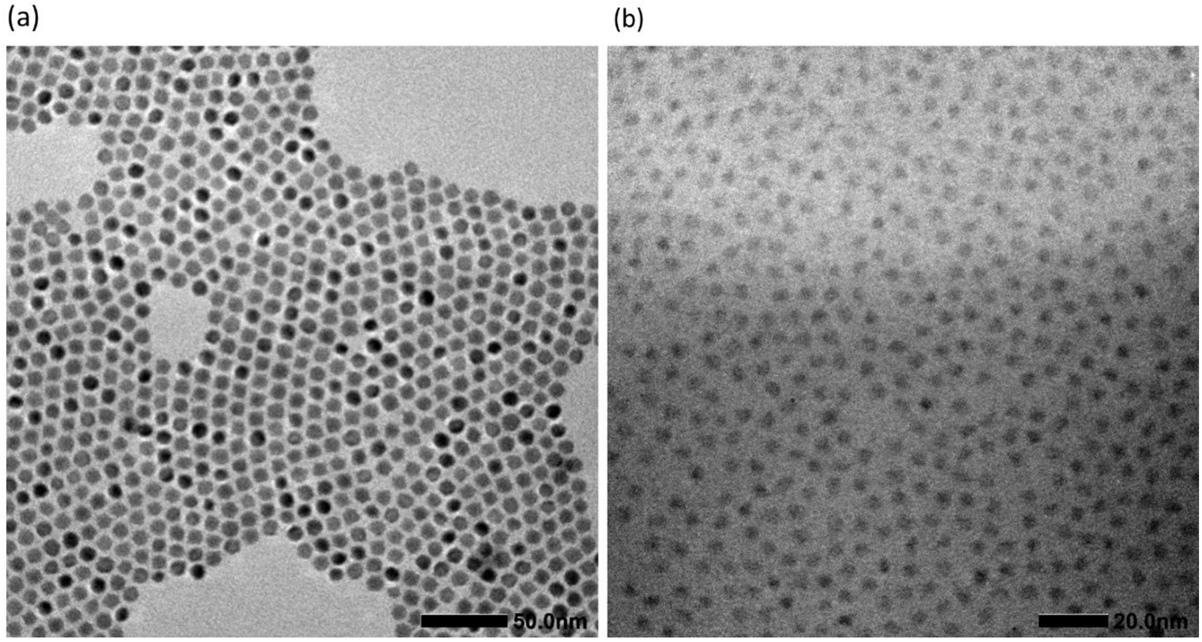

__Figure S1.__ (a) TEM image of 4 nm PbSe CQDs. (b) TEM image of 8 nm PbS CQDs.

2) Time Dependent XRD Measurements

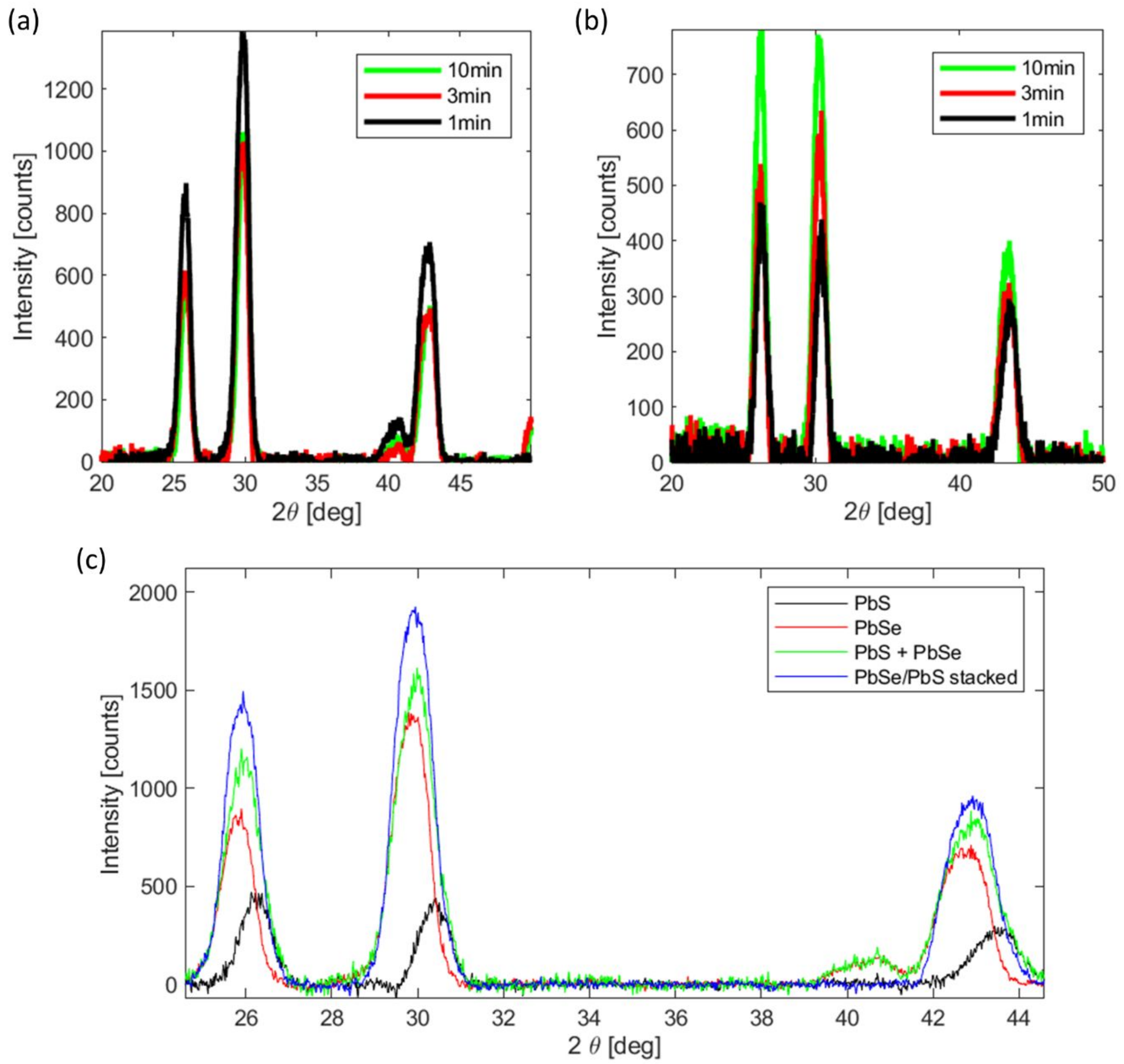

Figure S2. *(a) PbSe XRD spectra for different annealing times. It can be seen that the peak height decreases with increasing annealing time, indicating a decrease of crystallinity for longer annealing times. (b) PbS XRD spectra for different annealing times. It can be seen that the peak height increases with increasing annealing time, indicating an increase in crystallinity for longer annealing times. (c) XRD pattern a single layer of PbS (black) and a single layer of PbSe (red) annealed for 1 min at 310 °C. The arithmetic sum of the annealed PbSe and PbS pattern (green) and the XRD pattern of the stacked PbSe/PbS layers. It can be seen that the shape of the single sum of the single layers matches very well the shape of the PbSe/PbS bilayer stack. The difference in peak intensity is attributed to a difference in the thickness of the measured layers.*

3) AFM Characterization

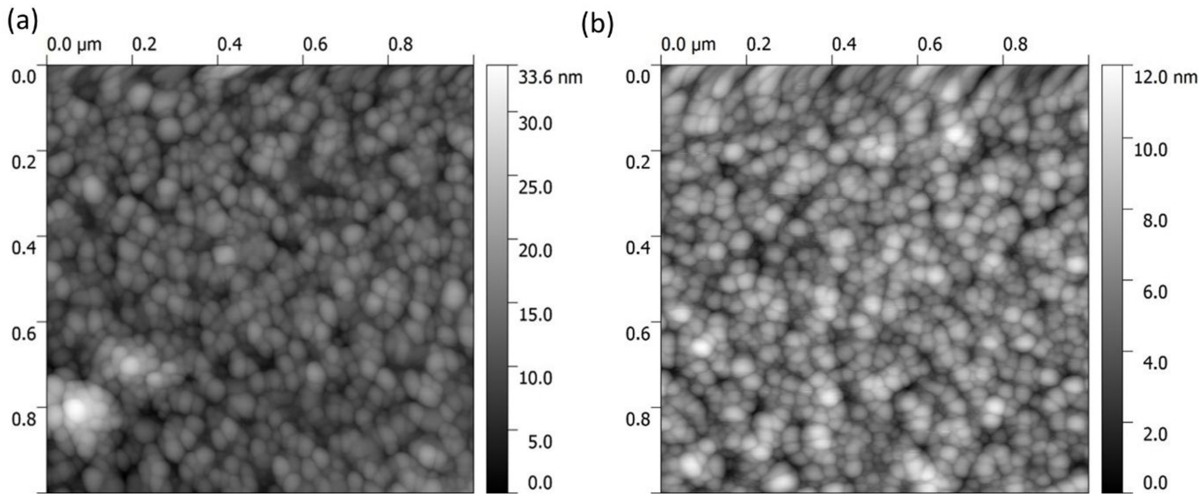

Figure S3. *(a) AFM image of 1 min at 310°C annealed PbSe showing a RMS surface roughness of 3.54 nm. (b) AFM image of 1 min at 310°C annealed PbS showing a RMS surface roughness 1.75nm.*

4) Optical Setup

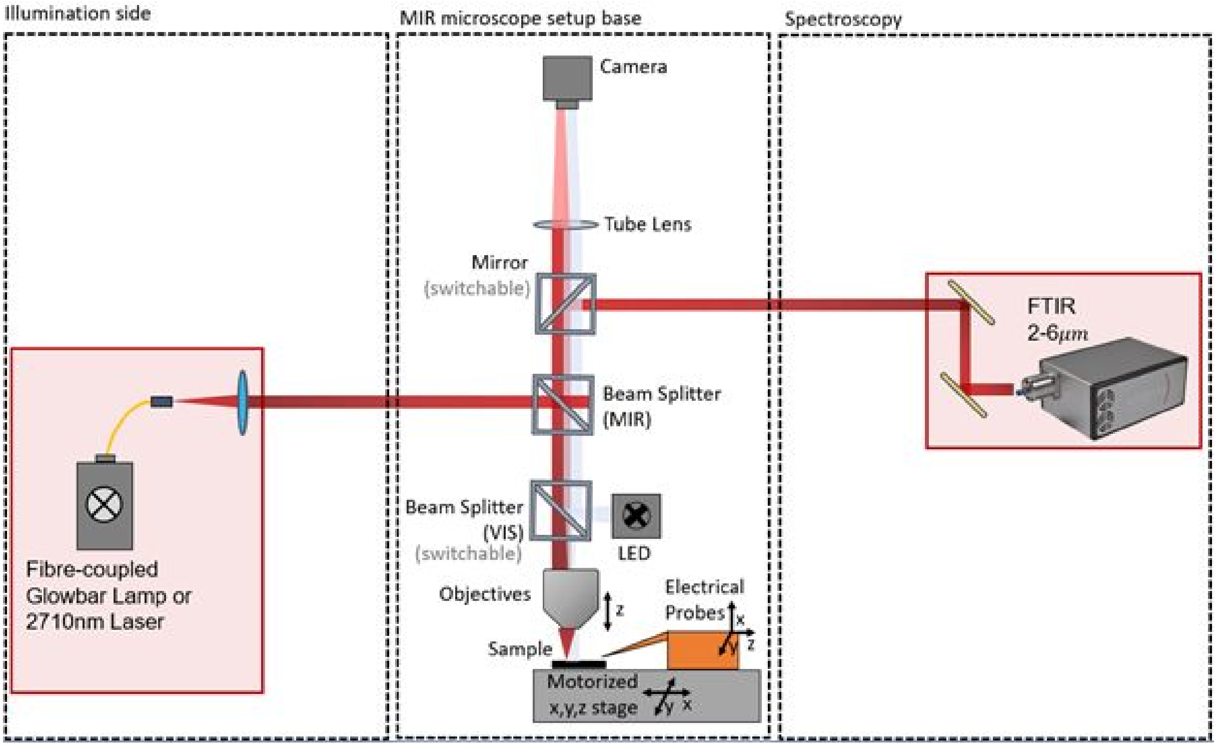

Figure S4. *Illustration of the optical setup used for the electro-optical characterization.*

5) Discussion on the Origin of the Photoconductivity in the Annealed PbSe CQD Layers

The annealed PbSe shows a significant photoresponse in the MIR without any sensitization. Recent works suggest that the formation of trap states at grain boundaries, which are often accredited to oxides, play a significant role for the photoconductance of PbSe [3,4]. Although, no oxides are expected to form in the annealed PbSe CQD layer, we suspect that trap states exist at the grain boundaries.

More precisely, layers consisting of CQDs have a very large surface due to the large surface to volume ratio of the individual CQDs. The surfaces of CQDs usually have a large number of defects which is why a lot of effort has been put into different ligand exchange methods to passivate the surface and surface traps. We expect that during the annealing most of ligands evaporate and several CQDs fuse into larger grains. Despite the fusing we suspect that a large number of the original traps on surface of the individual CQDs are still present at the grain boundaries of the annealed layer. Since the CQDs crystallize into small grains many boundaries with the accompanying traps exist in theses layers, which lead to an increased photoresponse.

6) Discussion on the Presences of a Heterojunction

In the main text we have already argued that the heterojunction formation can be observed by the fact that the current decreases in the PbSe/PbS stack when applying a forward current due to the depletion of carriers at the interface. Here we give more arguments for the formation of a heterojunction.

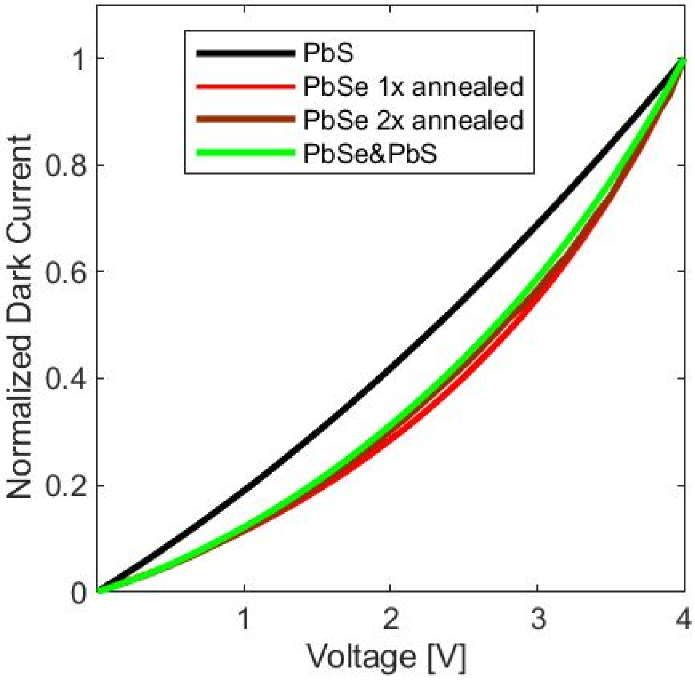

***Figure S5**. To maximum current normalized dark current. It can be seen that the different materials form different contact types to the Au electrodes.*

It can be expected that the two materials form a heterojunction since bulk PbSe has an electron affinity of $\chi_{PbSe,\,bulk} \approx 4.7\ \mathrm{eV}$ and a bulk band gap of $E_{g,\,PbSe} \approx 0.27\ \mathrm{eV}$, which is known to form a heterojunction with PbS ($\chi_{PbS,\,bulk} \approx 4.55\ \mathrm{eV}$, $E_{g,PbS} \approx 0.4\ \mathrm{eV}$) [5–8]. Although these values can differ for CQDs[7,9–11] and the exact values are unknown for the annealed CQD layers presented in this work, the formation of a heterojunction between individual PbS and PbSe crystal domains may be expected due to the differences in the bandgap.

A further indicator of the presence of a heterojunction can be seen by analyzing the normalized I-V curves shown in Figure S5. PbS forms an ohmic contact with Au which results in the nearly linear I-V, whereas PbSe forms a Schottky type contact. This difference in contact type indicates that the barrier height between Au and the pristine materials PbS and PbSe are likely to be different. In such a case it can be argued that energetic band position of PbS and PbSe differ as well, which also should lead to the formation of a heterojunction if the materials are brought into contact with each other.

7) Time Dependent Photoresponse

In Figure S6 (a) the photoresponse over a long period of time is shown and in Figure S6 (b) the photocurrent at a modulation frequency of 4 Hz, which was used to measure a responsivity of ~250 A/W. The ratio of the maximum current of these measurements was used to calculate the maximum responsivity of ~375 A/W at a wavelength of 2710nm. Both measurements were performed under identical illumination conditions.

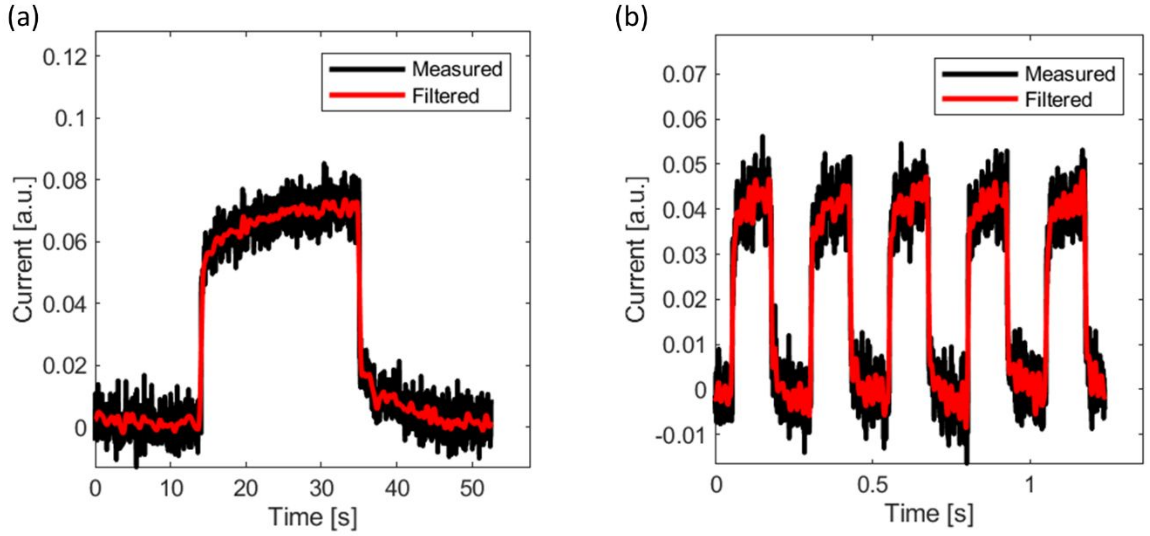

Figure S6. *(a) Time dependent photoresponse of PbSe/PbS metamaterial enhanced photodetector. (b) Time dependent photoresponse of PbSe/PbS metamaterial enhanced photodetector at a modulation frequency of 4 Hz. Both measurements were performed under identical illumination conditions at a wavelength of 2710 nm.*

8) Polarization Dependent Passive Absorption

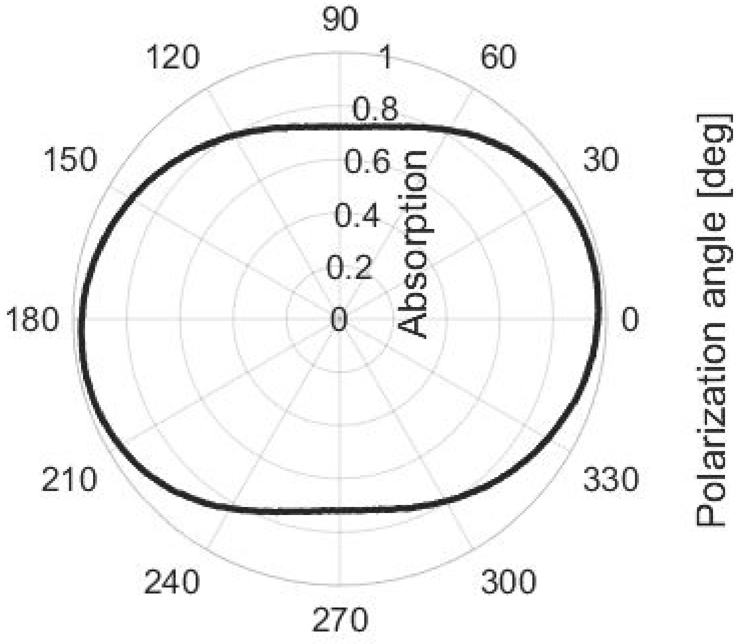

Figure S7. *Polarization dependent passive absorption of a PbSe/PbS metamaterial enhanced photodetector (l = 350nm and p = 1 µm) characterized at a wavelength of 2710 nm with a polarization controller. It can be seen that the absorption does not drop below 75% and that a near unity absorption can be reached for an ideal polarization state.*

9) Characterization Metamaterial Photodetectors

Table S1. *Metamaterial parameters and wavelengths at which they were characterized.*

Device	Characterization Wavelength [nm]	Resonator length *l* [nm]	Period *p* [µm]
**i**	2710	350	1
**ii**	3250	400	0.8
**iii**	4000	550	0.9
**iv**	4250	600	0.9

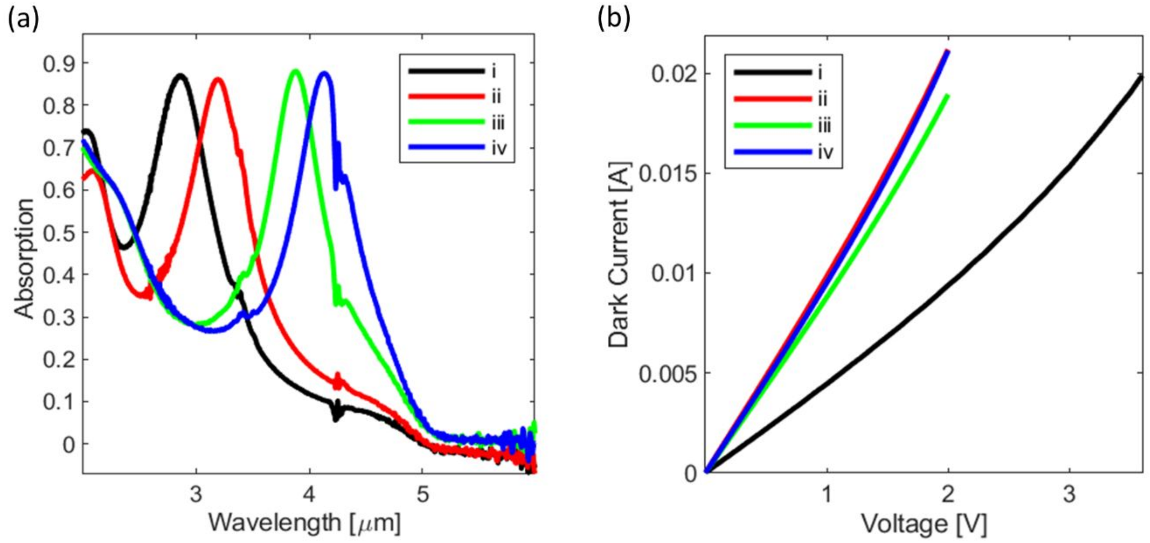

Figure S8. *Additional measurements of the metamaterial photodetectors. Design parameters of the different detectors are listed in Table S1. (a) Passive absorption spectra of metamaterial photodetectors. (b) Dark I-V measurements of metamaterial photodetectors.*

10) Comparison of Photodetectors

The below presented table provides an overview of recent publications of photodetectors operating in the MIR. The responsivities and detectivities are used as a figure of merit. This comparison focuses on 2D and CQD materials and their combination and is not complete. A single representative responsivity and detectivity value was selected if several values were given in the referenced publications

Table S2. *Comparison of this work and representative recent publications. Listing the operation wavelength, responsivity, detectivity, operating temperature and references.*

Material	Wavelength (nm)	Responsivities (A/W)	Detectivity [Jones]	Temperature (K)	REF
**PbSe, PbS & Metamaterial**	2710	375	1×10^{8}	290	this work
**PbSe, PbS& Metamaterial**	3250	208	5×10^{7}	290	this work
**PbSe, PbS& Metamaterial**	4000	16	3×10^{6}	290	this work
**PbSe, PbS& Metamaterial**	4250	7.85	9×10^{5}	290	this work
**HgTe**	2500	0.8	6.5×10^{11}	290	12
**HgTe**	4800	1.5	5.4×10^{10}	80	13
**HgTe & Metamaterial**	4500	1.62	4×10^{11}	85	14

**HgTe & Graphene**	4000	0.08	4.2×10^{10}	138	15
**HgTe & Graphene**	2500	800	4.2×10^{8}	80	16
**HgTe & Graphene**	2500	0.0065	3×10^{9}	290	17
**HgTe & MOS_2**	2100	5000	$1*10^{12}$	290	18
**HgSe**	6000	0.8	1.5×10^{8}	290	19
**HgSe**	5000	0.0013	3×10^{8}	80	20
**HgSe & Hg Te**	4400	0.001	1.5×10^{9}	RT	21
**Ag_2Se**	4500	0.02	1×10^{7}	RT	22
**Ag_2Se**	4400	0.013	3×10^{5}	RT	23
**PbSe**	3500	11	4.5×10^{10}	RT	24
**PbSe**	4000	49	-	RT	25
**PbSe**	4000	8	-	RT	26
**Graphene & metamaterial**	4000	0.036	5×10^{6}	RT	27
**Graphene & Ti_2O_3**	4200	300	7×10^{8}	RT	28
**Graphene & hBN antenna**	6600	0.027	-	RT	29
**Graphene & antenna**	3000	1	-	RT	30
**WS_2 & HfS_2**	4700	791	3×10^{10}	RT	31
**BP**	3400	0.518	-	77	32
**BP**	3390	82	-	RT	33
**BP & PETG**	4320	3	5.97×10^{9}	RT	34

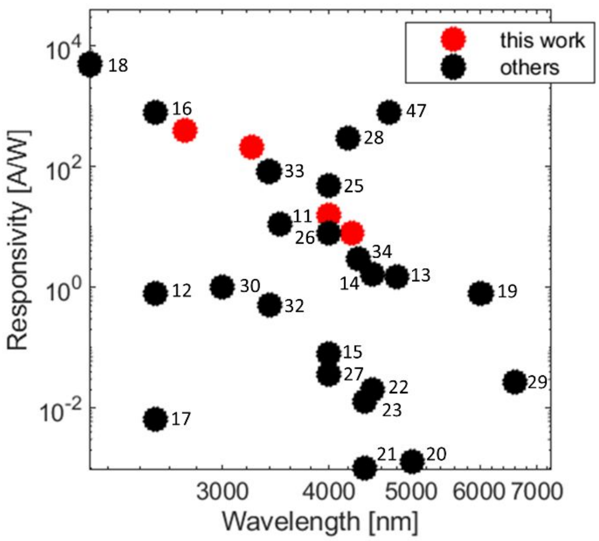

Figure S9. *Responsivity comparison of published works and this work with references.*

References

(1) Hines, M. A.; Scholes, G. D. Colloidal PbS Nanocrystals with Size-Tunable Near-Infrared Emission: Observation of Post-Synthesis Self-Narrowing of the Particle Size Distribution. *Adv. Mater.* **2003**, *15* (21), 1844–1849. https://doi.org/10.1002/adma.200305395.

(2) Luther, J. M.; Law, M.; Song, Q.; Perkins, C. L.; Beard, M. C.; Nozik, A. J. Structural, Optical, and Electrical Properties of Self-Assembled Films of PbSe Nanocrystals Treated with 1,2-Ethanedithiol. *ACS Nano* **2008**, *2* (2), 271–280. https://doi.org/10.1021/nn7003348.

(3) Harrison, J. T.; Gupta, M. C. Mechanistic Studies of Oxidation and Iodization of PbSe Thin Film Sensitization for Mid-Infrared Detection. *J. Appl. Phys.* **2022**, *131* (2). https://doi.org/10.1063/5.0077053.

(4) Humphrey, J. N.; Petritz, R. L. Photoconductivity of Lead Selenide: Theory of the Mechanism of Sensitization. *Phys. Rev.* **1957**, *105* (6), 1736–1740. https://doi.org/10.1103/PhysRev.105.1736.

(5) Wei, S. H.; Zunger, A. Electronic and Structural Anomalies in Lead Chalcogenides. *Phys. Rev. B - Condens. Matter Mater. Phys.* **1997**, *55* (20), 13605–13610. https://doi.org/10.1103/PhysRevB.55.13605.

(6) Knapp, R. A.; of Zhyst, D.; of Rochester, V.; Irorh, P. DE HAAS-SHUBNI KOV EFFE CT IN Sb Photoelectric Properties of Lead Sulfide in the Near and Vacuum Ultraviolet*. **1963**, *132* (5).

(7) Hyun, B. R.; Zhong, Y. W.; Bartnik, A. C.; Sun, L.; Abruña, H. D.; Wise, F. W.; Goodreau, J. D.; Matthews, J. R.; Leslie, T. M.; Borrelli, N. F. Electron Injection from Colloidal PbS Quantum Dots into Titanium Dioxide Nanoparticles. *ACS Nano* **2008**, *2* (11), 2206–2212. https://doi.org/10.1021/nn800336b.

(8) Dalven, R. A Review of the Semiconductor Properties of PbTe, PbSe, PbS and PbO. *Infrared Phys.* **1969**, *9* (4), 141–184. https://doi.org/10.1016/0020-0891(69)90022-0.

(9) Choi, J. J.; Lim, Y. F.; Santiago-Berrios, M. K. E. B.; Oh, M.; Hyun, B. R.; Sun, L.; Bartnik, A. C.; Goedhart, A.; Malliaras, G. G.; Abruña, H. D.; Wise, F. W.; Hanrath, T. PbSe Nanocrystal Excitonic Solar Cells. *Nano Lett.* **2009**, *9* (11), 3749–3755. https://doi.org/10.1021/nl901930g.

(10) Jasieniak, J.; Califano, M.; Watkins, S. E. Size-Dependent Valence and Conduction Band-Edge Energies of Semiconductor Nanocrystals. *ACS Nano* **2011**, *5* (7), 5888–5902. https://doi.org/10.1021/nn201681s.

(11) Miller, E. M.; Kroupa, D. M.; Zhang, J.; Schulz, P.; Marshall, A. R.; Kahn, A.; Lany, S.; Luther, J. M.; Beard, M. C.; Perkins, C. L.; Van De Lagemaat, J. Revisiting the Valence and Conduction Band Size Dependence of PbS Quantum Dot Thin Films. *ACS Nano* **2016**, *10* (3), 3302–3311. https://doi.org/10.1021/acsnano.5b06833.

(12) Ackerman, M. M.; Chen, M.; Guyot-Sionnest, P. HgTe Colloidal Quantum Dot Photodiodes for Extended Short-Wave Infrared Detection. *Appl. Phys. Lett.* **2020**, *116* (8), 083502. https://doi.org/10.1063/1.5143252.

(13) Chen, M.; Lan, X.; Tang, X.; Wang, Y.; Hudson, M. H.; Talapin, D. V.; Guyot-Sionnest, P. High Carrier Mobility in HgTe Quantum Dot Solids Improves Mid-IR Photodetectors. *ACS Photonics* **2019**, *6* (9), 2358–2365. https://doi.org/10.1021/acsphotonics.9b01050.

(14) Tang, X.; Ackerman, M. M.; Guyot-Sionnest, P. Thermal Imaging with Plasmon Resonance Enhanced HgTe Colloidal Quantum Dot Photovoltaic Devices. *ACS Nano* **2018**, *12* (7), 7362–7370. https://doi.org/10.1021/acsnano.8b03871.

(15) Guyot-Sionnest, P.; Roberts, J. A. Background Limited Mid-Infrared Photodetection with Photovoltaic HgTe Colloidal Quantum Dots. *Appl. Phys. Lett.* **2015**, *107* (25). https://doi.org/10.1063/1.4938135.

(16) Grotevent, M. J.; Hail, C. U.; Yakunin, S.; Bachmann, D.; Calame, M.; Poulikakos, D.; Kovalenko, M. V.; Shorubalko, I. Colloidal HgTe Quantum Dot/Graphene Phototransistor with a Spectral Sensitivity Beyond 3 Mm. *Adv. Sci.* **2021**, *8* (6). https://doi.org/10.1002/advs.202003360.

(17) Noumbé, U. N.; Gréboval, C.; Livache, C.; Chu, A.; Majjad, H.; Parra López, L. E.; Mouafo, L. D. N.; Doudin, B.; Berciaud, S.; Chaste, J.; Ouerghi, A.; Lhuillier, E.; Dayen, J. F. Reconfigurable 2D/0D p-n Graphene/HgTe Nanocrystal Heterostructure for Infrared Detection. *ACS Nano* **2020**, *14* (4), 4567–4576. https://doi.org/10.1021/acsnano.0c00103.

(18) Huo, N.; Gupta, S.; Konstantatos, G. MoS2–HgTe Quantum Dot Hybrid Photodetectors beyond 2 Mm. *Adv. Mater.* **2017**, *29* (17). https://doi.org/10.1002/adma.201606576.

(19) Lhuillier, E.; Scarafagio, M.; Hease, P.; Nadal, B.; Aubin, H.; Xu, X. Z.; Lequeux, N.; Patriarche, G.; Ithurria, S.; Dubertret, B. Infrared Photodetection Based on Colloidal Quantum-Dot Films with High Mobility and Optical Absorption up to THz. *Nano Lett.* **2016**, *16* (2), 1282–1286. https://doi.org/10.1021/acs.nanolett.5b04616.

(20) Chen, M.; Shen, G.; Guyot-Sionnest, P. Size Distribution Effects on Mobility and Intraband Gap of HgSe Quantum Dots. *J. Phys. Chem. C* **2020**, *124* (29), 16216–16221. https://doi.org/10.1021/acs.jpcc.0c05268.

(21) Livache, C.; Martinez, B.; Goubet, N.; Gréboval, C.; Qu, J.; Chu, A.; Royer, S.; Ithurria, S.; Silly, M. G.; Dubertret, B.; Lhuillier, E. A Colloidal Quantum Dot Infrared Photodetector and Its Use for Intraband Detection. *Nat. Commun.* **2019**, *10* (1). https://doi.org/10.1038/s41467-019-10170-8.

(22) Hafiz, S. Bin; Al Mahfuz, M. M.; Lee, S.; Ko, D. K. Midwavelength Infrared P-n Heterojunction Diodes Based on Intraband Colloidal Quantum Dots. *ACS Appl. Mater. Interfaces* **2021**, *13* (41), 49043–49049. https://doi.org/10.1021/acsami.1c14749.

(23) Hafiz, S. Bin; Al Mahfuz, M. M.; Ko, D. K. Vertically Stacked Intraband Quantum Dot Devices for Mid-Wavelength Infrared Photodetection. *ACS Appl. Mater. Interfaces* **2021**, *13* (1), 937–943. https://doi.org/10.1021/acsami.0c19450.

(24) Dortaj, H.; Dolatyari, M.; Zarghami, A.; Alidoust, F.; Rostami, A.; Matloub, S.; Yadipour, R. High-Speed and High-Precision PbSe/PbI2 Solution Process Mid-Infrared Camera. *Sci. Rep.* **2021**, *11* (1), 1533. https://doi.org/10.1038/s41598-020-80847-4.

(25) Dolatyari, M.; Rostami, A.; Mathur, S.; Klein, A. Trap Engineering in Solution Processed PbSe Quantum Dots for High-Speed MID-Infrared Photodetectors. *J. Mater. Chem. C* **2019**, *7* (19), 5658–5669. https://doi.org/10.1039/c8tc06093b.

(26) Dolatyari, M.; Rostami, A.; Mathur, S.; Klein, A. UV/IR Dual-Wavelength Photodetector Design Based on ZnO/PMMA/PbSe Nanocomposites. *IEEE Trans. Nanotechnol.* **2018**, *17* (3), 574–581. https://doi.org/10.1109/TNANO.2018.2827201.

(27) Wei, J.; Li, Y.; Wang, L.; Liao, W.; Dong, B.; Xu, C.; Zhu, C.; Ang, K. W.; Qiu, C. W.; Lee, C. Zero-Bias Mid-Infrared Graphene Photodetectors with Bulk Photoresponse and Calibration-Free Polarization Detection. *Nat. Commun.* **2020**, *11* (1). https://doi.org/10.1038/s41467-020-20115-1.

(28) Yu, X.; Li, Y.; Hu, X.; Zhang, D.; Tao, Y.; Liu, Z.; He, Y.; Haque, M. A.; Liu, Z.; Wu, T.; Wang, Q. J. Narrow Bandgap Oxide Nanoparticles Coupled with Graphene for High Performance Mid-Infrared Photodetection. *Nat. Commun.* **2018**, *9* (1), 1–8. https://doi.org/10.1038/s41467-018-06776-z.

(29) Castilla, S.; Vangelidis, I.; Pusapati, V. V.; Goldstein, J.; Autore, M.; Slipchenko, T.; Rajendran, K.; Kim, S.; Watanabe, K.; Taniguchi, T.; Martín-Moreno, L.; Englund, D.; Tielrooij, K. J.; Hillenbrand, R.; Lidorikis, E.; Koppens, F. H. L. Plasmonic Antenna Coupling to Hyperbolic Phonon-Polaritons for Sensitive and Fast Mid-Infrared Photodetection with Graphene. *Nat. Commun.* **2020**, *11* (1). https://doi.org/10.1038/s41467-020-18544-z.

(30) Cakmakyapan, S.; Lu, P. K.; Navabi, A.; Jarrahi, M. Gold-Patched Graphene Nano-Stripes for High-Responsivity and Ultrafast Photodetection from the Visible to Infrared Regime. *Light Sci. Appl.* **2018**, *7* (1), 2047–7538. https://doi.org/10.1038/s41377-018-0020-2.

(31) Lukman, S.; Ding, L.; Xu, L.; Tao, Y.; Riis-Jensen, A. C.; Zhang, G.; Wu, Q. Y. S.; Yang, M.; Luo, S.; Hsu, C.; Yao, L.; Liang, G.; Lin, H.; Zhang, Y. W.; Thygesen, K. S.; Wang, Q. J.; Feng, Y.; Teng, J. High Oscillator Strength Interlayer Excitons in Two-Dimensional Heterostructures for Mid-Infrared Photodetection. *Nat. Nanotechnol.* **2020**, *15* (8), 675–682. https://doi.org/10.1038/s41565-020-0717-2.

(32) Chen, X.; Lu, X.; Deng, B.; Sinai, O.; Shao, Y.; Li, C.; Yuan, S.; Tran, V.; Watanabe, K.; Taniguchi, T.; Naveh, D.; Yang, L.; Xia, F. Widely Tunable Black Phosphorus Mid-Infrared Photodetector. *Nat. Commun.* **2017**, *8* (1). https://doi.org/10.1038/s41467-017-01978-3.

(33) Guo, Q.; Pospischil, A.; Bhuiyan, M.; Jiang, H.; Tian, H.; Farmer, D.; Deng, B.; Li, C.; Han, S. J.; Wang, H.; Xia, Q.; Ma, T. P.; Mueller, T.; Xia, F. Black Phosphorus Mid-Infrared Photodetectors with High Gain. *Nano Lett.* **2016**, *16* (7), 4648–4655. https://doi.org/10.1021/acs.nanolett.6b01977.

(34) Kim, H.; Uddin, S. Z.; Lien, D. H.; Yeh, M.; Azar, N. S.; Balendhran, S.; Kim, T.; Gupta, N.; Rho, Y.; Grigoropoulos, C. P.; Crozier, K. B.; Javey, A. Actively Variable-Spectrum Optoelectronics with Black Phosphorus. *Nature* **2021**, *596* (7871), 232–237. https://doi.org/10.1038/s41586-021-03701-1.
